# Editorial: Placental physiology and placental derived stem cells for regenerative medicine

**DOI:** 10.3389/fphys.2023.1346913

**Published:** 2023-12-14

**Authors:** Jitendra K. Kanaujiya, Jay S. Mishra

**Affiliations:** ^1^ Alexion AstraZeneca Rare Disease, New Haven, CT, United States; ^2^ Department of Comparative Biosciences, School of Veterinary Medicine, University of Wisconsin-Madison, Madison, WI, United States

**Keywords:** artificial placenta, stem cells, intrauterine inflammation, preterm birth, regeneration, perfluorooctanoic acid (PFOA)

The maintenance of optimal placental physiology holds paramount importance for ensuring both balanced fetal growth and development, as well as the overall wellbeing of the mother during pregnancy. This significance arises from the placenta’s intricate role in facilitating the transfer of essential nutrients and other factors crucial for achieving successful pregnancy outcomes. By acting as a conduit for this exchange between the maternal and fetal circulatory systems, the placenta establishes and sustains a complex physiological equilibrium.

Beyond its immediate functions in supporting pregnancy, the human placenta emerges as a noteworthy source of stem cells with substantial implications for regenerative medicine. Stem cells derived from the placenta offer unique advantages, including the ability to foster optimal fetal growth and development. Importantly, the placenta’s role extends beyond its temporal function during pregnancy, as it serves as a reservoir of diverse types of stem cells.

The unique origin of placental tissues, originating in the early stages of embryonic development, raises intriguing possibilities. It suggests that these tissues may house cells retaining the plasticity observed in early embryonic cells, potentially unlocking therapeutic applications in regenerative medicine. The accessibility of the placenta in abundant quantities further enhances its appeal, and the non-intrusive nature of harvesting stem cells from this organ positions placenta-derived stem cells as highly attractive candidates for various regenerative medicine applications. This combination of factors underscores the promising potential of utilizing placental resources to advance therapeutic interventions in the field of regenerative medicine.

In this special edition, we present four articles that cover a wide range of topics related to placental physiology, intrauterine inflammation, and gestational exposure to perfluorooctanoic acid (PFOA). Two of the included studies assessed the efficacies of artificial placenta in the maintenance of preterm birth in ovine and pig models. The other two studies focused on analyzing the effect of PFOA exposure and intrauterine inflammation on placental development.

The study by Usuda et al. demonstrates the effective preservation of key physiological and hemodynamic parameters for up to 2 weeks in extremely preterm ovine fetuses, utilizing an artificial placenta platform and pharmacological intervention. Notably, this was achieved without any noticeable signs of infection and the study reveals a deviation in the growth trajectory of fetuses treated with artificial placenta technology when compared to the *in-utero* control group. This study represents a significant instance of an artificial placenta platform’s successful application in sustaining extremely preterm fetuses. The weight of the preterm fetus in this study closely aligns with that of human fetuses reaching the threshold of viability, typically observed at 21–24 weeks of gestation. In spite few instances of bleeding, during urachal catheterization, the findings of this study underscore the critical step forward toward enhancing the outcomes of extremely preterm births through artificial placental technology.

In the second article of this issue, Charest-Pekeski et al. present findings on the support of preterm fetal pigs, utilizing a circuit comprising an oxygenator and a centrifugal pump. Cannulation of umbilical vessels was performed, and the fetuses were sustained for a significantly longer duration on the pumped artificial placenta pump circuit, compared to the pumpless artificial placenta circuit. Additionally, upon the initiation of artificial placenta support with the pumped system, authors observed supraphysiological circuit flows, tachycardia, and hypertension, contrasting with the subphysiological flows exhibited by animals maintained on the pumpless artificial placenta circuit. The findings indicate that the incorporation of a centrifugal pump into the artificial placenta circuit enhances the survival of preterm pigs by augmenting umbilical veins flow through the reduction of right ventricular afterload. However, it is noteworthy that despite this improvement, the development of heart failure persisted within a matter of days. These observations shed light on the intricate dynamics involved in enhancing preterm survival through the utilization of artificial placenta systems.

Jiang et al. examined the gestational exposure of Perfluorooctanoic acid (PFOA) on Placental development. PFOA is a broadly used perfluorinated compound in industrial and consumer fields which is known to cause developmental toxicity (increased resorbed embryo, reduced fetal survival and growth retardation). In this article authors investigated the effect of PFOA exposure on placental development. For this author treated pregnant mice with different doses of PFOA and observed that PFOA exposure reduced weight of early placenta. Histological examination of placenta shows that PFOA exposure increases spongiotrophoblast and labyrinth area. Immunohistochemical staining demonstrated that PFOA exposure caused the shutting of fetal vessels and downregulation of uNK cell numbers in the deciduas of placenta. Finally, authors showed that PFOA exposure lead to apoptosis of placenta by upregulation of apoptotic genes Bax, cleaved caspase 3 and drastic change of nucleus morphology of placental cells. This study shows the potential risk of PFOA exposure in pregnant women and fetus development.

Spontaneous preterm birth (SPTB) is a global problem which lead to neonatal morbidity and mortality. SPTB complication is not well understood. Infection is reported in 70% of extreme SPTB cases. In their article Lien et al. showed that intrauterine inflammation affect the transcriptome and metabolome in placenta, which ultimately affects the placenta function that lead to SPTB. In this study authors used LPS induced mouse model of intrauterine infection and explored transcriptome and metabolome of placenta. Transcriptome profiling leads to identification of genes involved in various pathways such as vascular function and reactivity, increase oxidative stress and mitochondrial dysfunction, altered glucose and lipid metabolism, and affect upstream regulators and regulatory networks which are crucial for nutrient sensing and mitochondrial function. Metabolomics analysis resulted in identification of metabolic pathways that altered by infection of placenta such as increase in lipid metabolite Acylcarnitines, affect glucose metabolism, increased branched chain amino acid catabolism, decrease TCA cycle metabolites, increase Purine and reduce Pyrimidine catabolism, increased nicotinamide metabolism and altered choline metabolism. Transcriptomics and metabolomics data significantly overlap and provide greater insight into the mechanism of intrauterine inflammation in SPTB.

Altogether the articles collected in this Research Topic shed light on new advancements in artificial placenta technology in sustaining preterm fetuses in ovine and pig models, the effect of PFOA exposure on placental development, and the effect of intrauterine inflammation on transcriptome and metabolome of the placenta. Considering the critical role of the placenta in successful pregnancy and fetal development, these studies provide noteworthy advancements in the understanding of placental physiology and preterm fetal survival.

